# The Influence of Phacoemulsification on Surgical Outcomes of Trabeculectomy with Mitomycin-C for Uveitic Glaucoma

**DOI:** 10.1371/journal.pone.0151947

**Published:** 2016-03-18

**Authors:** Asaho Nishizawa, Toshihiro Inoue, Saori Ohira, Eri Takahashi, Junji Saruwatari, Keiichiro Iwao, Hidenobu Tanihara

**Affiliations:** 1 Department of Ophthalmology, Faculty of Life Sciences, Kumamoto University, Kumamoto, Japan; 2 Department of Pharmacology and Therapeutics, Faculty of Life Sciences, Kumamoto University, Kumamoto, Japan; Casey Eye Institute, UNITED STATES

## Abstract

**Purpose:**

To evaluate the influence of phacoemulsification after trabeculectomy on the postoperative intraocular pressure (IOP) in eyes with uveitic glaucoma (UG).

**Setting:**

Kumamoto University Hospital, Kumamoto, Japan.

**Design:**

A retrospective cohort study.

**Methods:**

The medical records of patients with UG who had trabeculectomy with mitomycin-C (MMC) were reviewed. Complete and qualified surgical failures were defined by an IOP of ≥21 mmHg (condition A), ≥18 mmHg (condition B), or ≥15 mmHg (condition C) without and with glaucoma eye drops, respectively. Kaplan-Meier survival analysis, generalized by the Wilcoxon test, and the Cox proportional hazards model analysis were conducted. Post-trabeculectomy phacoemulsification was treated as a time-dependent variable. In 24 (30%) of the included 80 eyes, phacoemulsification was included, and they were divided into two groups: groups I (8 eyes with phacoemulsification within 1 year after trabeculectomy) and group II (16 eyes after 1 year following trabeculectomy).

**Results:**

Multivariable Cox proportional hazards model analysis showed post-trabeculectomy phacoemulsification was a significant factor in both complete success and qualified success based upon condition C (*P* = 0.0432 and *P* = 0.0488, respectively), but not for the other conditions. Kaplan–Meier survival analyses indicated significant differences in success probabilities between groups I and group II for complete success and qualified success based upon condition C (*P* = 0.020 and *P* = 0.013, respectively). There was also a significant difference for qualified success based upon condition B (*P* = 0.034), while there was no significant difference for the other conditions.

**Conclusion:**

Post-trabeculectomy phacoemulsification, especially within 1 year, can cause poor prognosis of IOP control of UG eyes after trabeculectomy with MMC.

## Introduction

Intraocular pressure (IOP) reduction has been regarded as the main target for the management of glaucoma. Among a number of therapeutic modalities, trabeculectomy with mitomycin-C (MMC) has been regarded as an effective surgical therapy for glaucoma treatment. To date, some risk factors, such as a history of intraocular surgeries, juvenile and secondary glaucomas [including uveitic glaucoma (UG) and neovascular glaucoma], have been associated with the long-term failure of trabeculectomy, resulting in IOP re-elevation [1].

Phacoemulsification is required in some cases after trabeculectomy because non-physiological aqueous humor outflow causes the onset and progression of cataracts [2], [3], [4]. In our previous studies, we reported that a history of phacoemulsification is associated with poor prognosis for trabeculectomy with MMC for treatment of open-angle glaucoma [5], [6]. In addition, we reported that phacoemulsification (especially within 1 year after trabeculectomy) is significantly related to the long-term failure of trabeculectomy and to resultant elevated IOP levels in eyes with open-angle glaucoma [7]. It is possible that induced inflammatory responses and an altered microenvironment after phacoemulsification cause closure of the filtration route of the aqueous humor and/or the disappearance of filtration blebs created by trabeculectomy. Indeed, we reported elevated levels of proinflammatory cytokines, such as monocyte chemoattractant protein and interleukin-8, in the aqueous humor after phacoemulsification, and their effects on surgical results of trabeculectomy in open-angle glaucoma [8], [9]. However, uveitis and its associated inflammatory reactions are also regarded as risk factors for long-term failure of trabeculectomy [10]. In the present study, we show that the history of cataract surgery is associated with surgical failure, even in eyes with UG, and that UG eyes experience more frequent cataract surgeries after trabeculectomy than primary open-angle glaucoma (POAG) eyes. Thus, in these UG cases, phacoemulsification is required in addition to successful trabeculectomy, and postoperative inflammation could potentially affect control of the IOP. Consistent with this possibility, combined trabeculectomy with phacoemulsification has been reported to be associated with higher risks of surgical failure [11]. To our knowledge, however, no study has statistically evaluated the role of phacoemulsification on surgical results of trabeculectomy in eyes with UG.

We therefore report the influence of phacoemulsification, analyzed as a time-dependent factor, on surgical outcomes of trabeculectomy with MMC in eyes with UG, utilizing the Cox proportional hazards model as a method for analyzing risk factors.

## Patients and Methods

### Patients

This retrospective study was approved by the Institutional Review Board of Kumamoto University, and written informed consent was obtained if the participant was accessible. Prior to analysis, all patient records/information was anonymized and de-identified by a person independent of the authors. TI, KI, and HT were directly involved in the treatment of the patients included in this study, and JS analyzed data independently from the clinicians. All procedures adhered to the Declaration of Helsinki. Included in this retrospective review of medical records were 80 Japanese patients (80 eyes) with UG who underwent uncombined trabeculectomy with MMC at the Kumamoto University Hospital between April, 1996 and September, 2013. When both eyes of one patient fulfilled the criteria, we included only the eye that was treated first during the period of our analyses.

### Surgical methods and postoperative management

Trabeculectomy and postoperative management were performed as previously described [5]. Briefly, a conjunctival flap (fornix-based in 64 eyes, limbal-based in 9 eyes, and unknown in the remaining 7 eyes, Table 1) and a half-thickness scleral flap were created, and then exposed to MMC at 0.4 mg/mL for 4 min, followed by washing with 200 mL of balanced salt solution. A corneoscleral block was excised, and peripheral iridectomy was performed. The scleral flap and conjunctiva were closed with 10–0 monofilament nylon sutures. Postoperatively, all patients were prescribed similar topical medical regimens of 1% (w/v) topical atropine sulfate for 1 week, and 0.1% (w/v) topical betamethasone and topical antibiotics for approximately 3 months. The surgeons who performed the primary trabeculectomies considered the need for postoperative glaucoma eye drops based on the IOP values, the extent of visual field disturbances, and the appearance of blebs using slit lamp biomicroscopy. When the IOPs could not be controlled postoperatively by the maximum permissible dose of eye drops, additional glaucoma surgeries were performed.

**Table 1:** Baseline Characteristics of All 80 Patients (80 Eyes).

Characteristic	W/ phaco	With phaco	*P* value
Gender (%)			0.3464^a^
No. of women	25 (44.6)	8 (33.3)	
No. of men	31 (55.4)	16 (66.7)	
Age (years)			0.0247^b^
Mean ± SD	53.7 ± 16.3	61.9 ± 9.8	
Range	16 to 83	39 to 76	
Etiology of uveitis (%)			0.8478^a^
Granulomatous	19 (33.9)	8 (33.3)	
Non-granulomatous	7 (12.5)	2 (8.3)	
Unknown	30 (53.6)	14 (58.3)	
Follow-up period (months)			0.0740^b^
Mean ± SD	51.4 ± 48.6	72.0 ± 40.9	
Range	0.2 to 225.6	14.5 to 195.0	
Preoperative IOP (mm Hg)			0.3971^b^
Mean ± SD	35.9 ± 9.1	37.8 ± 8.2	
Range	15.7 to 58.7	22.0 to 51.5	
Type of phacoemulsification incision (%)			N/A
Corneal	N/A	14 (58.3)	
Scleral	N/A	6 (25.0)	
Unknown	N/A	4 (16.7)	
Base of conjunctival flap of TLE (%)			0.6041^a^
Fornix-based	46 (82.1)	18 (75.0)	
Limbal-based	5 (8.9)	4 (16.7)	
Unknown	5 (8.9)	2 (8.3)	

IOP, intraocular pressure; phaco, phacoemulsification; SD, standard deviation; TLE, trabeculectomy; W/O, without. P values were determined by means of χ square test^a^ or unpaired two-tailed t-test^b^. Significant at *P* < 0.05.

Phacoemulsification and postoperative management were performed as previously described [7]. Briefly, a 2.4–3.0 mm grooved incision was made at the temporal cornea or sclera while the filtering bleb was avoided. After the completion of the continuous curvilinear capsulorhexis and hydrodissection procedures, the lens nucleus was removed by phacoemulsification. Subsequently, the lens cortex was aspirated by irrigation/aspiration. The capsular bag was filled with sodium hyaluronate, a foldable intraocular lens was implanted in the capsular bag, and the sodium hyaluronate was removed from the anterior chamber and capsular bag. Antimetabolite injections were not given. All eyes were treated with antibiotics and steroid eye drops postoperatively.

### Main outcome measures

According to the guidelines from the World Glaucoma Association [12], we defined surgical success as an IOP of <21 mmHg (criterion A), <18 mmHg (criterion B), or <15 mmHg (criterion C) with (qualified success) or without (complete success) the use of topical ocular hypotensive medication. Follow-up was censored upon the performance of any additional non-glaucoma related surgery, such as vitrectomy, except for phacoemulsification. Complete failure was defined as a requirement for additional glaucoma surgery (including needling procedures) and hypotony of <4 mmHg. For this definition of surgical failure, the IOP obtained ≥2 months after trabeculectomy was used to avoid the effect of short-term postoperative IOP fluctuations. IOP levels were measured with a Goldmann applanation tonometer. The preoperative IOP for each eye was determined as the average of three measurements before trabeculectomy.

### Statistical methods

Data analyses were performed using the R software, version 3.1.1 (R Foundation for Statistical Computing). To assess the potential prognostic factors for surgical failure, the multivariable adjusted hazard ratio (HR) was determined using a Cox proportional hazards model, with six covariates at baseline involving mean pre-trabeculectomy IOP, the conjunctival flap procedure during trabeculectomy (fornix-based or limbal based), the period of medication for uveitis, the period of medication for glaucoma and granulomatous uveitis, and a time-dependent covariate (*i*.*e*., phacoemulsification). The cumulative incidence of surgical failure was plotted based on the Kaplan–Meier method, and the comparisons of cumulative incidences among patients with and without phacoemulsification were performed using the generalized Wilcoxon test. In the comparisons of cumulative incidences, the patients with and without the phacoemulsification were stratified according to the time interval between trabeculectomy and phacoemulsification (*i*.*e*., within or more than 1 year). A *P*-value less than 0.05 was considered statistically significant.

## Results

**Fig 1:**
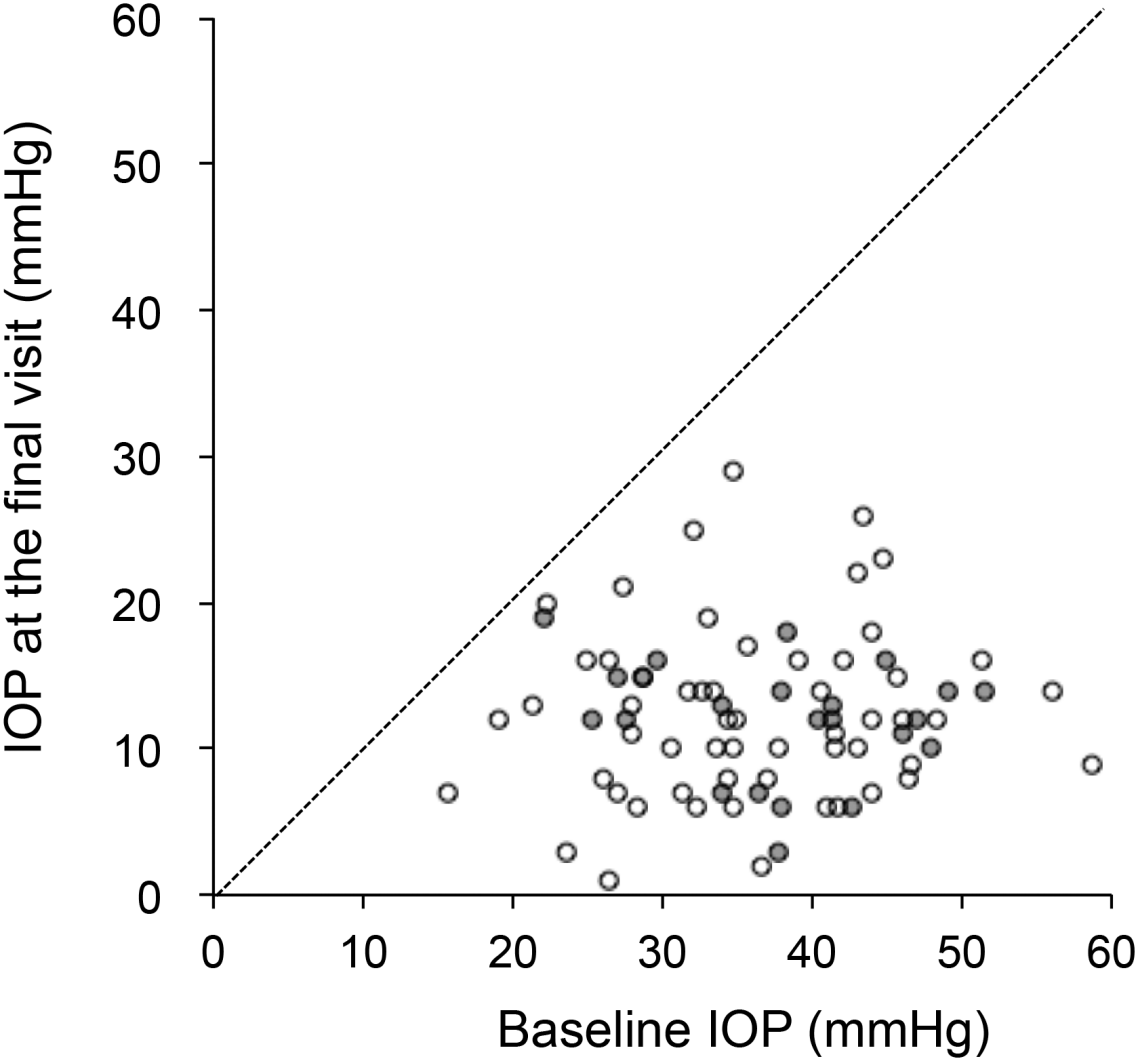
Scatter diagram of the baseline pre-trabeculectomy intraocular pressure (IOP) and IOP at the last follow-up. White and grey dots represent data of eyes without and with post-trabeculectomy phacoemulsification, respectively.

In the present study, 80 eyes (80 patients) satisfied the inclusion criteria. The mean IOP (± standard deviation) at the last visit (12.3 ± 5.4 mmHg) was significantly lower compared with the baseline IOP (36.4 ± 8.8 mmHg; *P* < 0.001; Fig 1), and the mean number of glaucoma eye drops was significantly smaller (0.7 ± 1.2 versus 2.9 ± 0.7; *P* < 0.001). In 24 (30%) of the 80 eyes, post-trabeculectomy phacoemulsification was added. Patient characteristics categorized by the presence of post-trabeculectomy phacoemulsification are shown in Table 1. Age was significantly younger in patients with post-trabeculectomy phacoemulsification (*P* = 0.0247). The mean (± standard deviation) interval between the trabeculectomy and phacoemulsification was 779 ± 1,162 days (range, 170–5,607 days), and the mean pre-phacoemulsification IOP was 10.4 ± 3.9 days (range, 2.7–17.7 mmHg). In 8 (33%) of the 24 eyes, phacoemulsification was performed within 1 year after trabeculectomy. The etiologies of the uveitis are listed in Table 2.

**Table 2:** Etiology of Uveitis of All 80 Patients (80 Eyes).

Etiology of uveitis (%)	W/O phaco	With phaco
Sarcoidosis	12 (21.4)	4 (16.7)
Posner-Schlossman syndrome	5 (8.9)	1 (4.2)
Vogt-Koyanagi-Harada disease	3 (5.4)	2 (8.3)
VZV-associated anterior uveitis	2 (3.6)	0
HLA-B27 associated anterior uveitis	1 (1.8)	1 (4.2)
Acute retinal necrosis	1 (1.8)	0
Toxoplasmosis	1 (1.8)	0
HSV-associated anterior uveitis	0	1 (4.2)
HTLV-1-associated uveitis	0	1 (4.2)
Unknown	31 (55.4)	14 (58.3)

HTLV, human T-cell leukemia virus; HSV, herpes simplex virus; phaco, phacoemulsification; VZV, varicella zoster virus; W/O, without.

The Cox proportional hazards model was used to evaluate the risk of potential prognostic factors for the results of trabeculectomy. Included factors were post-trabeculectomy phacoemulsification as a time-dependent variable, age, sex, pre-trabeculectomy IOP, duration of uveitis therapy, duration of glaucoma therapy, and granulomatous uveitis. As shown in Tables 3 and 4, post-trabeculectomy phacoemulsification was a significant factor in both complete success and qualified success based upon condition C (*P* = 0.0432 and *P* = 0.0488, respectively), but not for the other conditions (Tables 5–8).

**Table 3:** Results of the Cox Proportional Hazards Model Analysis for Condition C (Complete Success).

Variable	RR	95% CI	*P* value^a^
Phacoemulsification (time-dependent variable)	3.37	1.04 to 10.09	0.043
The type of uveitis (granulomatous vs. non-granulomatous)	1.35	0.32 to 5.65	0.687
Age (per year)	0.99	0.96 to 1.02	0.482
Male vs. female	0.88	0.41 to 1.90	0.743
Pre-trab IOP (per mm Hg)	1.01	0.97 to 1.05	0.687
Duration of uveitis therapy (within 1 year vs. after 1 year before trab)	1.02	0.42 to 2.43	0.974
Duration of glaucoma therapy (within 1 year vs. after 1 year before trab)	0.86	0.38 to 1.94	0.716

CI, confidence interval; IOP, intraocular pressure; RR, relative risk; trab, trabeculectomy; vs., versus.

^a^ P values were determined by means of the Wald test in the Cox proportional hazards model.

Significant at P < 0.05.

**Table 4:** Results of the Cox Proportional Hazards Model Analysis for Condition C (Qualified Success).

Variable	RR	95% CI	*P* value^a^
Phacoemulsification (time-dependent variable)	3.32	1.01 to 10.93	0.049
The type of uveitis (granulomatous vs. non-granulomatous)	1.38	0.33 to 5.82	0.664
Age (per year)	0.99	0.96 to 1.02	0.464
Male vs. female	0.84	0.38 to 1.82	0.645
Pre-trab IOP (per mm Hg)	1.01	0.97 to 1.06	0.568
Duration of uveitis therapy (within 1 year vs. after 1 year before trab)	0.97	0.41 to 2.33	0.948
Duration of glaucoma therapy (within 1 year vs. after 1 year before trab)	0.86	0.38 to 1.94	0.719

CI, confidence interval; IOP, intraocular pressure; RR, relative risk; trab, trabeculectomy; vs., versus.

^a^ P values were determined by means of the Wald test in the Cox proportional hazards model.

Significant at P < 0.05.

**Table 5:** Results of the Cox Proportional Hazards Model Analysis for Condition A (Complete Success).

Variable	RR	95% CI	*P* value^a^
Phacoemulsification (time-dependent variable)	1.51	0.45 to 5.10	0.510
The type of uveitis (granulomatous vs. non-granulomatous)	2.13	0.40 to 11.41	0.377
Age (per year)	1.00	0.97 to 1.03	0.868
Male vs. female	0.94	0.36 to 2.43	0.892
Pre-trab IOP (per mm Hg)	0.96	0.91 to 1.01	0.114
Duration of uveitis therapy (within 1 year vs. after 1 year before trab)	1.01	0.34 to 2.99	0.993
Duration of glaucoma therapy (within 1 year vs. after 1 year before trab)	0.95	0.35 to 2.64	0.928

CI, confidence interval; IOP, intraocular pressure; RR, relative risk; trab, trabeculectomy; vs., versus.

^a^ P values were determined by means of the Wald test in the Cox proportional hazards model.

Significant at P < 0.05.

**Table 6:** Results of the Cox Proportional Hazards Model Analysis for Condition A (Qualified Success).

Variable	RR	95% CI	*P* value^a^
Phacoemulsification (time-dependent variable)	1.21	0.27 to 5.36	0.807
The type of uveitis (granulomatous vs. non-granulomatous)	2.70	0.34 to 21.22	0.345
Age (per year)	0.99	0.96 to 1.03	0.690
Male vs. female	1.04	0.34 to 3.23	0.941
Pre-trab IOP (per mm Hg)	0.97	0.91 to 1.03	0.329
Duration of uveitis therapy (within 1 year vs. after 1 year before trab)	0.71	0.20 to 2.50	0.591
Duration of glaucoma therapy (within 1 year vs. after 1 year before trab)	0.97	0.29 to 3.23	0.960

CI, confidence interval; IOP, intraocular pressure; RR, relative risk; trab, trabeculectomy; vs., versus.

^a^ P values were determined by means of the Wald test in the Cox proportional hazards model.

Significant at P < 0.05.

**Table 7:** Results of the Cox Proportional Hazards Model Analysis for Condition B (Complete Success).

Variable	RR	95% CI	*P* value^a^
Phacoemulsification (time-dependent variable)	2.05	0.70 to 6.04	0.193
The type of uveitis (granulomatous vs. non-granulomatous)	1.46	0.33 to 6.49	0.620
Age (per year)	0.99	0.97 to 1.02	0.612
Male vs. female	0.84	0.37 to 1.89	0.671
Pre-trab IOP (per mm Hg)	0.98	0.94 to 1.03	0.449
Duration of uveitis therapy (within 1 year vs. after 1 year before trab)	0.82	0.34 to 1.96	0.656
Duration of glaucoma therapy (within 1 year vs. after 1 year before trab)	0.96	0.40 to 2.30	0.922

CI, confidence interval; IOP, intraocular pressure; RR, relative risk; trab, trabeculectomy; vs., versus.

^a^ P values were determined by means of the Wald test in the Cox proportional hazards model. Significant at P < 0.05.

**Table 8:** Results of the Cox Proportional Hazards Model Analysis for Condition B (Qualified Success).

Variable	RR	95% CI	*P* value^a^
Phacoemulsification (time-dependent variable)	1.39	0.43 to 4.48	0.581
The type of uveitis (granulomatous vs. non-granulomatous)	2.54	0.44 to 14.48	0.295
Age (per year)	0.99	0.96 to 1.02	0.429
Male vs. female	1.28	0.54 to 3.05	0.579
Pre-trab IOP (per mm Hg)	1.00	0.95 to 1.05	0.895
Duration of uveitis therapy (within 1 year vs. after 1 year before trab)	0.54	0.21 to 1.36	0.188
Duration of glaucoma therapy (within 1 year vs. after 1 year before trab)	0.94	0.36 to 2.44	0.900

CI, confidence interval; IOP, intraocular pressure; RR, relative risk; trab, trabeculectomy; vs., versus.

^a^ P values were determined by means of the Wald test in the Cox proportional hazards model.

Significant at P < 0.05.

**Fig 2:**
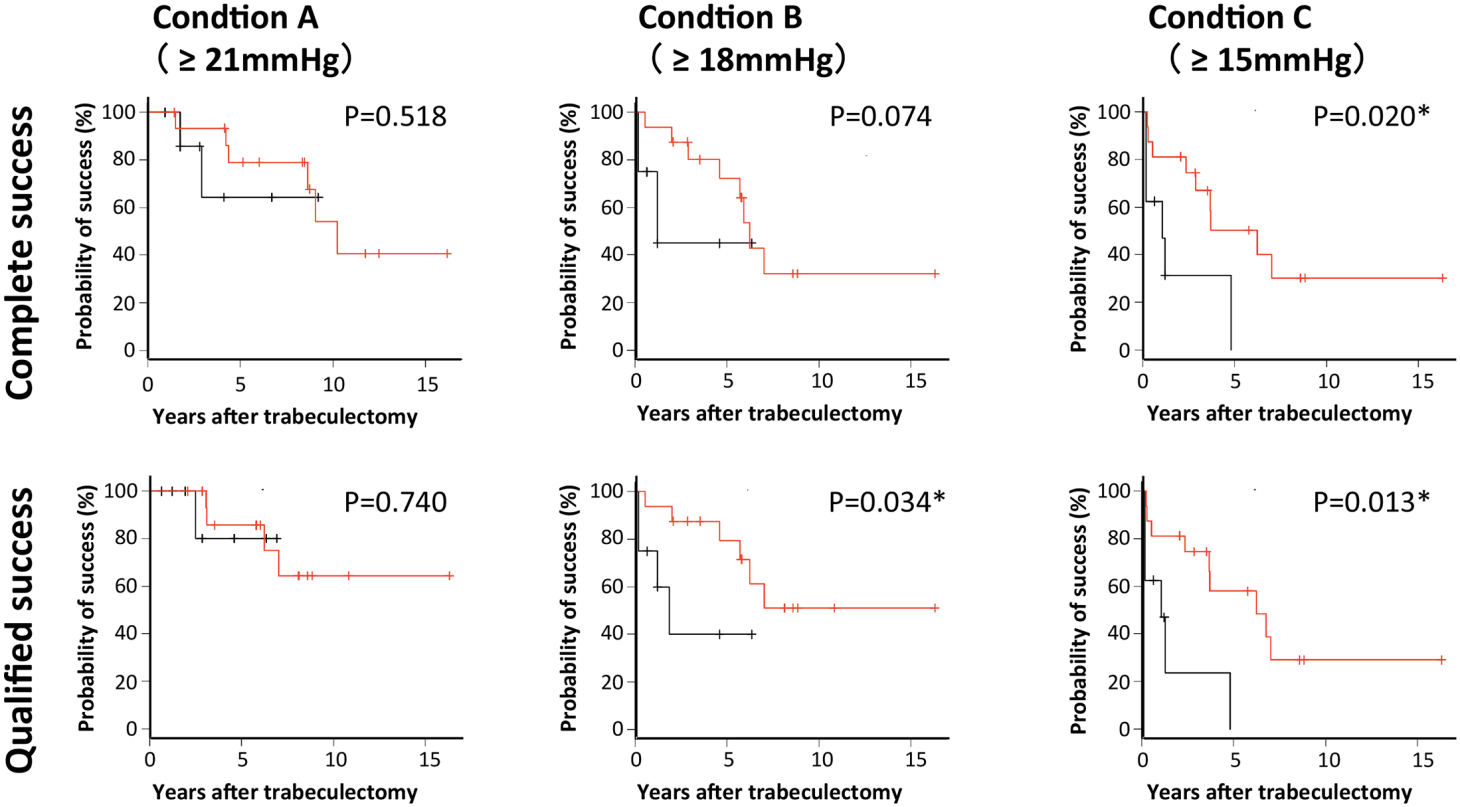
Kaplan–Meier survival curves for the probability of surgical success over time in 16 patients who were treated with phacoemulsification more than 1 year after trabeculectomy phacoemulsification (red lines), and 8 patients who were treated with phacoemulsification within 1 year after trabeculectomy (black lines). *P < 0.05, generalized Wilcoxon test.

Because it was a time-dependent variable, post-trabeculectomy phacoemulsification, was identified as a prognostic factor for the surgical success of trabeculectomy. The time between trabeculectomy and phacoemulsification has been considered a key factor for post-trabeculectomy IOP control. Thus, we divided the patients with phacoemulsification into two groups: 8 eyes with phacoemulsification within 1 year after trabeculectomy (group I) and the remaining 16 eyes after 1 year following trabeculectomy (group II), then conducted Kaplan–Meier survival analyses for all conditions. Generalized Wilcoxon tests showed significant differences (*P* = 0.020 and *P* = 0.013, respectively) for complete success and qualified success based upon condition C between groups I and II (Fig 2). In addition, there was a significant difference for qualified success based upon condition B (*P* = 0.034), while there was no significant difference for the other conditions.

## Discussion

In UG cases, postoperative inflammation, previous cataract surgery, an etiology of uveitis, combined surgery with phacoemulsification, omission of MMC, intraocular inflammation at surgery, postoperative inflammation, male sex, and younger age were reported to be associated with poor IOP control after trabeculctomy [10], [11]. [13], [14]. In the present study, post-trabeculectomy phacoemulsification, especially within 1 year after trabeculectomy, was shown to be a prognostic factor for failure to keep the IOP below 15 mmHg after trabeculectomy with MMC. To our knowledge, this is the first report that assesses the effect of phacoemulsification on IOP control after trabeculectomy. In our present study, 24 (30%) of the 80 eyes were treated with phacoemulsification post-trabeculectomy. In contrast, 37 (21%) of the 178 eyes with open-angle glaucoma required phacoemulsification post-trabeculectomy in a previous study [7]. Because the mean follow-up period was longer for the UG cases (57 ± 47 months) compared with open-angle cases (37 ± 32 months), it is possible that the cataract progressed more in UG cases than open-angle cases. Alternatively, there were factors associated with uveitis that induced cataract progression. Consistent with this possibility, UG eyes after trabeculectomy required more cataract surgery than the POAG eyes.^10^ Taken together, the results suggest that increased attention should be directed to the progression of cataract and the timing of phacoemulsification in eyes with UG.

The impact of post-trabeculectomy phacoemulsification on IOP control has been of interest to clinicians. However, this impact has not been easy to assess due to the following reasons. First, prospective randomized comparative studies are not acceptable for most patients because the visual loss from cataract progression causes severe inconvenience in their daily lives. In addition, there is a long follow-up period to wait for the progression of cataract after trabeculectomy, and the assessment of post-phacoemulsification IOP control lengthens the follow-up period. For example, the mean interval period between trabeculectomy and phacoemulsification was 779 ± 1,162 days (range, 170–5,607 days) in the present study. Thus, a prospective study design may not be possible to answer this clinical question. Second, simple grouping based upon the presence of post-trabeculectomy phacoemulsification is not adequate to assess the effects of surgery, because elderly patients undergo cataract surgery more frequently compared with younger patients. Moreover, lower IOP is reportedly a risk factor for the progression of cataract after trabeculectomy [4]. Thus, the group with a history of post-trabeculectomy phacoemulsification would be older with better IOP control compared with its control group. Considering that young age is a well-known prognostic factor for trabeculectomy results, the comparison of success probabilities between the groups categorized by the presence of post-trabeculectomy phacoemulsification may not accurately assess the impact of the surgery on IOP control. Additionally, this method does not consider the time period between trabeculectomy and phacoemulsification, which was reportedly a prognostic factor [7]. Finally, a comparison of IOP values before and after phacoemulsification cannot answer this clinical question, because IOP values are sometimes elevated during follow-up periods without any ocular surgery. Thus, we employed the multivariable Cox hazards model, in which post-trabeculectomy phacoemulsification was treated as a time-dependent factor, together with a retrospective study. This method considers the time period between the surgeries, plus other background factors including age.

Multivariate analysis showed that post-trabeculectomy phacoemulsification as a time-dependent variable was a significant risk factor for both complete and qualified success for condition C (*P* = 0.043 and *P* = 0.048, respectively). In addition, the success probability for group I (phacoemulsification within 1 year after trabeculectomy) was significantly worse than group II (phacoemulsification after 1 year following trabeculectomy) for both complete and qualified success for condition C (*P* = 0.020 and *P* = 0.013, respectively). There was also a significant difference between the groups for qualified success for condition B (*P* = 0.034). This finding is consistent with our previous results on the negative impact of phacoemulsification on surgical outcomes in eyes with POAG and exfoliation glaucoma [7]. Taken together, the results suggest that the addition of phacoemulsification (especially within a short period following trabeculectomy) can cause an elevation in the IOP.

Because postoperative inflammation was significantly associated with poor IOP control [11], [13], [14], the negative impact of post-trabeculectomy phacoemulsification on trabeculectomy results may involve post-phacoemulsification inflammation. During inflammation, the aqueous humor contains high levels of proinflammatory cytokines and cells [15], [16], [17], [18], [19], [20], [21] that can cause a wound healing reaction in the bleb, resulting in surgical failure. In our previous study, the aqueous level of monocyte chemotactic protein-1, one of the major proinflammatory cytokines, was associated with the width of filtration opening on the scleral flap created at an early stage by trabeculectomy [22], and affected the surgical results of trabeculectomy [9]. Thus, adequate control of post-phacoemulsification inflammation may be important in achieving better IOP control in UG eyes with a history of trabeculectomy.

The present study showed that a significant difference was not detected for granulomatous UG cases when compared with other causes. This finding contradicts our previous report on poor prognosis of trabeculectomy in granulomatous UG cases [10]. The difference may be caused by the differences in the statistical power based on the sample number between the studies; 204 cases were included in the past study, while 80 cases were included in the present study. Thus, we were unable to make any conclusions regarding the associations of the uveitis diagnoses from the present study. The small sample number is a limitation of this study. Another limitation was that the present study was conducted in a single center that may have resulted in selection bias. In addition, unknown factors that affect both cataract progression and trabeculectomy results may exist, so the association between post-trabeculectomy phacoemulsification and the poor IOP control could be indirect.

In conclusion, the present study demonstrates that post-trabeculectomy phacoemulsification, especially within 1 year, can cause poor prognosis of IOP control of UG eyes after trabeculectomy with MMC.
